# *In-silico* design, expression, and purification of novel chimeric *Escherichia coli* O157:H7 OmpA fused to LTB protein in *Escherichia coli*

**DOI:** 10.1371/journal.pone.0173761

**Published:** 2017-03-15

**Authors:** Aytak Novinrooz, Taghi Zahraei Salehi, Roya Firouzi, Sina Arabshahi, Abdollah Derakhshandeh

**Affiliations:** 1 Department of Pathobiology, School of Veterinary Medicine, Shiraz University, Shiraz, Iran; 2 Department of Microbiology and Immunology, Faculty of Veterinary Medicine, University of Tehran, Tehran, Iran; Beijing Institute of Microbiology and Epidemiology, CHINA

## Abstract

*E*. *coli* O157:H7, one of the major EHEC serotypes, is capable of developing bloody diarrhea, hemorrhagic colitis (HC), and fatal hemolytic uremic syndrome (HUS) and is accompanied by high annual economic loss worldwide. Due to the increased risk of HC and HUS development following antibiotic therapy, the prevention of infections caused by this pathogen is considered to be one of the most effective ways of avoiding the consequences of this infection. The main aim of the present study was to design, express, and purify a novel chimeric protein to develope human vaccine candidate against *E*. *coli* O157:H7 containing loop 2–4 of *E*. *coli* O157:H7, outer membrane protein A (OmpA), and B subunit of *E*. *coli* heat labile enterotoxin (LTB) which are connected by a flexible peptide linker. Several online databases and bioinformatics software were utilized to choose the peptide linker among 537 analyzed linkers, design the chimeric protein, and optimize the codon of the relative gene encoding this protein. Subsequently, the recombinant gene encoding OmpA-LTB was synthesized and cloned into pET-24a (+) expression vector and transferred to *E*. *coli* BL21(DE3) cells. The expression of OmpA-LTB chimeric protein was then carried out by induction of cultured *E*. *coli* Bl21 (DE3) cells with 1mM isopropyl-β-D-thiogalactopyranoside (IPTG). The purification of OmpA-LTB was then performed by nickel affinity chromatography. Expression and purification were analyzed by sodium dodecyl sulphate poly acrylamide gel electrophoresis. Moreover, the identity of the expressed protein was analyzed by western blotting. SDS-PAGE and western immunoblotting confirmed the successful expression of a 27 KDa recombinant protein after 24 hours at 37°C post-IPTG induction. OmpA-LTB was then successfully purified, using nickel affinity chromatography under denaturing conditions. The yield of purification was 12 mg per liter of culture media. Ultimately, we constructed the successful design and efficient expression and purification of OmpA-LTB divalent under the above-mentioned conditions.

## Introduction

The Enterohemorrhagic *Escherichia coli*, EHEC, which is a well-known foodborne and waterborne pathogen, evolves from nonpathogenic *E*. *coli* by acquiring a number of virulence factors. EHEC consists of different serotypes such as O157:H7 which are implicated human illnesses. *E*. *coli* O157:H7 is recognized as a cause of bloody diarrhea and hemorrhagic colitis (HC) and, in few cases, may lead to fatal hemolytic uremic syndrome (HUS), especially in young children [1–2]. Since the early 1980s, the adverse impact of *E*. *coli* O157:H7 infection has been regarded as a major public health and a threat of economic loss, particularly in developed countries [3–6]. According to CDC reports, O157:H7 is one of the four major foodborne pathogens causing diarrhea [7]. This pathogen is mainly transmitted to human population through the consumption of contaminated food products, including uncooked meat, unpasteurized dairy products, and vegetables contaminated by cattle feces. Since this serotype is a part of normal flora of cattle intestinal tract, 'the infected livestock shed these bacteria in high levels in their faces. In addition, the infectious dose of this pathogen is believed to be as low as 10 to 100 cells. Thus, eliminating this infection from the environment and human population appears to be very hard to achieve [2, 8–9]. Due to the high prevalence of resistance to antibacterial agents among *Enterobacteriaceae*, the antibiotic therapy may lead to failure in eliminating this infection from humans. Furthermore, this category of treatment for *E*. *coli* O157:H7 infections is controversial due to the risk of HUS development [2, 10–11]. Hence, in order to prevent the outbreaks of this infection, vaccination is the only significant alternative. Following the identification of *E*. *coli* O157:H7 specific virulence factors such as type III secretory system effector proteins and Shiga toxin, many efforts are being made to develop potential vaccines based on the antigenicity of these serotype specific virulence factors [12–16]. The first step in the pathogenicity of *E*. *coli* O157:H7 is adherence and colonization of the bacteria in the human intestine. Certain adhesion factors such as outer membrane protein A, or OmpA, and intimin play significant roles in colonization of this bacteria and initiation of pathogenesis. However, thus far, no effective vaccine has been available to prevent the initial adhesion of O157:H7 to intestinal epithelial cells and stop the initiation of the disease. OmpA is a well-studied conserved protein among *E*. *coli* serotypes which is structurally involved in cell stability besides its crucial role in pathogenicity of certain *E*. *coli* serotypes such as O157:H7. Several reports have demonstrated the role of OmpA in the initial adhesion of the bacteria as well as its ability to stimulate pro-inflammatory cytokines, IL-1, and IL-10. On the other hand, monoclonal antibodies against OmpA inhibit EHEC adherence to epithelial cells [17–20]. In point of fact, fact, the unique and specific structure of four-surface hydrophilic loops exposed to the N-terminal of OmpA enables this protein to induce host-innate and adoptive immune responses [21–23]

Heat-labile enterotoxin (LT) is the main virulence factor of enterotoxigenic *Escherichia coli* that causes traveler’s diarrhea in humans [24]. LT has a hexameric structure consisting of one unique A subunit (LTA) and five identical B subunits (LTB). LTB shows a great affinity to GM1 gangliosides located on the cell surfaces of a wide range of mammalian cells, including intestinal epithelial cells and Peyer’s patches. It is also proved that LTB is indirectly involved in LT delivery to the intestinal cells [25]. Therefore, it appears that LTB is capable of being accumulated on the Peyer’s patches and delivering any conjugated antigen or drug to the mesenteric macrophages and B lymphocytes through these patches. Immunogenic properties of LTB have been previously demonstrated by triggering lymphocytes and directing Th1 and Th2 responses [13, 26–27]. Orally administrated pentameric LTB is reported to be a non-toxic polypeptide [28]. Hence, this protein appears to be a safe component to be employed in designing vaccine for in vivo experiments. The interesting stability of EtxB_5_ quaternary structure in acidic and basic environments (pH = 2.0 to pH = 11.0) [25] facilitates and insures the effectiveness of its oral administration. Following that, the co-administration of a nontoxic adjuvant protein such as LTB with an immunogenic protein such as OmpA appears to be capable of prompting a strong immune response against the immunogenic protein, OmpA, and probably both proteins.

The aim of the current research was to design, express, and purify chimeric OmpA-LTB in order to introduce appropriate vaccine candidate to combat EHEC infections in human. To the best of our knowledge, chimeric OmpA-LTB is the first chimeric derivative of OmpA protein produced and used for any biological and medical purposes.

## Materials and methods

### In-silico design of chimeric protein

In order to design the chimeric OmpA-LTB, OmpA loop 2–4 encoding sequence was selected to be linked to LTB coding sequence. Therefore, nucleotide 181–576 of full length *ompA* (NCBI accession number NC_002655.2), was fused to nucleotide 64–372 of *eltb* (NCBI accession number: NC_014232.1). To construct the chimeric protein in a way that resembles the native folding and function of both OmpA loop 2–4 and LTB, an appropriate peptide linker was designed to join the above-mentioned regions of OmpA and LTB together. Totally, 537 natural and synthetic linkers from two protein linker databases (http://www.ibi.vu.nl/programs/linkerdbwww and http://parts.igem.org/Protein_domains/Linker) were analyzed to identify the most suitable linker which maintains the native folding of the recombinant protein. Briefly, three dimensional structures of chimeric proteins, each containing one linker, were obtained as a PDB (protein data bank) file by introducing amino acid sequences of chimeric proteins to Protein Homology/analogY Recognition Engine (Phyre2) (http://www.sbg.bio.ic.ac.uk/~phyre2/html/page.cgi?id=index). Afterwards, residue-to-residue alignments of 3D structures of chimeric proteins with native OmpA loop 2–4 and LTB were performed, using TM-align to analyze the similarity of 3D structure of relevant proteins based on the TM-score achieved (http://zhanglab.ccmb.med.umich.edu/TM-align) [29]. The amino acid sequence of chimeric protein containing the selected linker was then converted back to nucleotide sequence by an in-silico sequence conversion tool (http://in-silico.net/tools/biology/sequence_conversion). The nucleotide sequence of the recombinant gene was codon-optimized for being expressed in *E*. *coli*, using OPTIMIZER (http://genomes.urv.es/OPTIMIZER). The *E*. *coli* B strain codon usage table was adapted from a codon usage database (www.kazusa.or.jp/codon). The codon adaptation index (CAI), which has a direct relationship with the expression level of the desired gene, was analyzed by both OPTIMIZER and Gene Script rare codon analysis tool (http://www.genscript.com/cgibin/tools/rare_codon_analysis) to determine the expression efficiency of the recombinant gene in *E*. *coli* B strains.

### Plasmid construction

To generate OmpA-LTB chimeric protein, the 3’ end of the selected *ompA* fragment was joined to 5’ end of the *eltb* gene through a nucleotide sequence encoding a G-S-G-S-G-S peptide linker. The gene encoding mentioned in the chimeric protein was synthesized and cloned into a commercial pET-24a (+) vector by Biomatik (Cambridge, Canada) to generate OmpA-LTB-pET24a plasmid. OmpA-LTB-pET24a plasmid contains a sequence which encodes a hexa-histidine tag at the 3’ end to allow further purification of chimeric protein by Nickel affinity columns. 5’ and 3’ ends of the above-mentioned sequence were flanked by NdeI and XhoI digestion sites, respectively. OmpA-LTB-pET24a plasmid was transformed into *E*. *coli* DH5 α cells on LB agar plates containing 50μg/ml kanamycin. The transformed colonies were analyzed by polymerase chain reaction, using T7 promoter and T7 terminator primers and an enzymatic digestion using the above restriction enzymes to validate the insert size and orientation. Cultures of the validated colonies were preserved at -80°C for further applications.

### Expression and purification of recombinant protein

OmpA-LTB-PET24 plasmid was also transformed into *E*. *coli* BL21 (DE3) cells. A transformed clone harboring OmpA-LTB-PET24 plasmid was inoculated in 1 liter LB broth containing kanamycin (50μg/ml), and the culture was incubated at 37°C under aerobic condition and shaking at 250 rpm. The expression of the cultured cells was induced as soon as the cells reached the log phase (optical density of the culture having reached 0.7–0.8) by 1mM IPTG (isopropyl-β-D-thiogalactopyranoside) (sinaclon, Tehran, Iran). One ml of the cultured cells was collected from the uninduced cells just before the addition of IPTG (for further use as negative control) and from induced cells at 1, 2, 3, 4, and 24-hour-post-induction. The collected cultures were centrifuged to harvest the bacterial pellets, and the pellets were stored at -20°C for further use.

The bacterial pellets were collected from 1 liter of 24-hour-post-induction LB broth cultures at the above-mentioned conditions for purification of chimeric protein by HisPur^™^ Ni-NTA Purification Kit (Thermo scientific, Rockford, USA). The purification of 6x his-tagged OmpA-LTB protein was performed under denaturing condition. Lysis buffer (7M Urea, 100mM NaH_2_PO_4_, 10mM Tris-CL, pH 8.0) was added to and mixed with cell pellets, then incubated at room temperature for 1 hour, and centrifuged at 14000g for 30 minutes. The supernatant was utilized for purification by Ni-NTA affinity chromatography, using the user’s prepared wash buffer (8M Urea, 100mM NaH_2_PO_4_, 10mM Tris-CL, pH 6.3) and elution buffer (8M Urea, 100mM NaH_2_PO_4_, 10mM Tris-CL, pH 4.5). Finally, the purity of the eluted protein was analyzed on 4–12% Sodium Dodecyl Sulphate Polyacylamide Gel Electrophoresis, SDS-PAGE. The purified chimeric protein was precipitated using cold absolute ethanol, washed and resuspended in 1ml phosphate-buffered saline (PBS).

### SDS-PAGE and western blot analysis

The bacterial pellets were lysed by 50 μl 2x sample buffer (0.125M Tris-base pH 6.8, 4% SDS, 20% glycerol, 0.5% bromophenol blue, and 10% 2-Mercaptoethanol) and heated at 100°C for 10 minutes. Eventually, the prepared samples were collected, and the expression of the recombinant protein was analyzed by 4–12% SDS PAGE. The whole cell lysate proteins were transferred into nitrocellulose membrane (Sigma-Aldrich) for western blotting analysis by Trans-Blot SD Semi-Dry Transfer cell system (Biorad, USA). Subsequent to the western transfer of proteins, nitrocellulose membrane was blocked in TBS solution containing 4% skimmed milk to block nonspecific antibody binding overnight. Afterwards, the nitrocellulose membrane was washed with TBS and incubated with HRP conjugated mouse anti-polyhistdine monoclonal antibody (Sigma-Aldrich, St. Louis, MO, USA) in 4% skimmed milk solution, 1:1000, for 1 hour at room temperature. Finally, the blotting reaction was performed, using diaminobenzidine (DAB)/H2O2 (Sigma-Aldrich, Poole, UK).

### Ethics statement

This study was approved by Animal Ethics Committee (AECs) of Faculty of Veterinary Medicine, University of Tehran, and all the animal experiments were performed in accordance with regulations of this committee.

### Animal

A total number of five inbred Six- to eight-week-old female BALB/c mice (26-28g) were housed in plastic cages with stainless steel wire lids and fed with a standard pellet diet and water ad libitum. Mice were maintained under standard temperature conditions (21 ± 1°C) with a controlled 12 h:12 h light–dark cycles. Following the sera collection, mice were humanely euthanized by exposure to CO_2_. All the animal experiments performed in this study were conducted according to standard animal ethic guidelines with considering animal welfare behavior and protecting them from pain and discomfort provided by Animal Ethics Committee (AECs) of Faculty of Veterinary Medicine, University of Tehran in compliance with the Helsinki Declaration.

### Animal immunization

The BALB/c mice (26-28g) were injected subcutaneously with 0.1 ml of recombinant OmpA-LTB (50μg) with Equal amount of Freund’s complete adjuvant in the first dose. Subsequently, same dose of this chimeric protein was emulsified with Equal amount of Freund’s incomplete adjuvant and injected 10 days later. The negative control mouse was injected only with 100 μl PBS. Then, the sera of mice were collected 11 days after the last antigen administration.

### Dot-blot immunoassay

The dot-blot immunoassay was performed on nitrocellulose membrane (Sigma-Aldrich). Recombinant protein OmpA-LTB was spotted on the membrane by placing 1 μl of antigen solution (12mg/ml in PBS). The membrane was air-dried for 15 minutes at room temperature. Then it was blocked in 5% nonfat dried milk in TBS at 37°C for 2 hours with gentle shaking. After blocking, the dotted regions of membrane were incubated for 1 hour with mouse serum which is considered as primary antibody. After washing the membrane with TBS, 2 μl of 0.5 mg/ml FITC anti-mouse IgG (BioLegend, San Diego, USA) was added to the dotted regions of membrane and incubated for 1 hour at 37°C. The membrane was washed twice with TBS and visualized by a U.V transiluminator (Labnet, Edison, NJ, USA).

## Results and discussion

Since ruminants, especially healthy cattle are the main reservoir of this serotype, spreading contamination in farms and dairy products are inevitable. In spite of excreting high amount of organism in calves’ faces, colonization and organism shedding are asymptomatic unlike in human. *Escherichia coli* serotype O157:H7 develops severe form of EHEC disease in human by causing bloody diarrhea, hemolytic anemia, HC and kidney damages. Therefore, it is vital to develop strategies to combat EHEC disease in human. Due to frequent antibiotic resistance phenotypes among EHECs and high risk of HUS development following the antibiotic therapy, one of the best strategies to prevent and control the outbreaks of EHEC O157:H7 infections is vaccination [30–31]. Although many attempts have been made to produce an effective vaccine to eradicate O157:H7 infection [12–13,15–16, 32], no effective vaccine for human is available to confront this foodborne pathogen. Adherence of *E*. *coli* O157:H7 to host intestinal epithelium is considered as the first stage of disease initiation. Several studies have confirmed OmpA as a critical adhesion and have supported its significant role in this initial phase [17–18]. Outer membrane protein A has been recognized as an antigenic protein associated with pathogenic features of this bacterium, i.e. initial adhesion, serum and antimicrobial peptide resistance, intracellular survival, and its invasion to the host cells. Due to the spatial location of four epitopes of this protein on the cell surface, it can easily stimulate innate and adoptive immune responses [18–21]. *E*. *coli* and *Shigella flexneri* OmpA-mediated activation of dendritic cells and macrophages followed by producing cytokines have been previously described [18–20, 33]. The extensive antibody response to OmpA of *E*. *coli* O157:H7 and other *Enterobacteriacea* species has been ascertained by many studies [19–20, 34–36]. Hence, capability of OmpA to induce antibody responses brings up this protein as a good vaccine candidate. It is obvious this multifunctional outer membrane protein is highly conserved among *Enterobacteriaceae*. Thus, antibody responses against OmpA would not limit to EHEC strains and Cross protection against pathogenic serotypes of *Enterobacteriacea* might be occured [19]. Although, antibody responses against OmpA reduce adherence of EHEC and other related strains, but; it definitely does not have any impact on colonization of non-pathogenic *E*. *coli*, present in intestinal tract of human as normal flora [37–40].

OmpA N-terminal epitopes are essential for stabling and correctly folding this antigenic protein. The potential virulent effects of each recombinant OmpA loop have been reported previously. Recombinant loops 1–3, 2–3, and 2–4 of OmpA are capable of protecting mice from experimental *E*. *coli* intracerebral infection [23]. In our study, a chimeric protein consisted of loop 2–4 of *E*. *coli* O157:H7 OmpA, a GSGSGS linker; furthermore, enterotoxigenic *E*. *coli* LTB was designed and expressed successfully. The linker was employed to achieve the highest similarity between 3D structure of this recombinant protein and that of the native OmpA and LTB.

### In-silico design and plasmid construction

**Fig 1:**
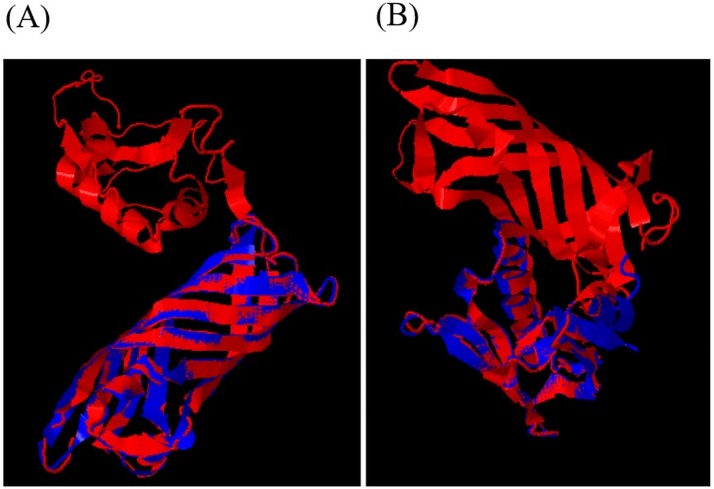
3D structure alignment of chimeric protein. Panel (A): Alignment of OmpA-LTB (in red) with native OmpA (in Blue). Panel (B): 3D structure alignment of OmpA-LTB (in red) with native LTB (in Blue).

537 combinations of chimeric OmpA-LTB amino acid sequence were generated by using 537 different peptide linkers. The amino acid sequences were submitted to phyre2 software, and 3D structures of recombinant proteins were downloaded as PBD files. The 3D structures of 537 chimeric proteins were compared with those of native LTB and loop 2–4 OmpA by TM-align software. This software exhibited the rate of similarity index between the recombinant proteins and their native structure by measuring TM-score. The TM-score varies in the range of 0–1. TM-score of 1 indicates a perfect similarity between the 3D structures of the two proteins. All scores above 0.5 indicate an acceptable similarity between the two structures; additionally, the scores below 0.2 show a random similarity between the structures of the two proteins. The highest TM-scores were calculated as 0.92 in topology comparison of OmpA-LTB with native LTB and 0.97 in topology comparison of OmpA-LTB with native loop 2–4 OmpA (Fig 1). The above-mentioned TM-scores are related to a recombinant OmpA-LTB in which a GSGSGS linker connects the C-terminus of OmpA to the N-terminus of LTB. Thus, among the analyzed linkers, a GSGSGS peptide was determined as the most suitable one.

**Fig 2:**
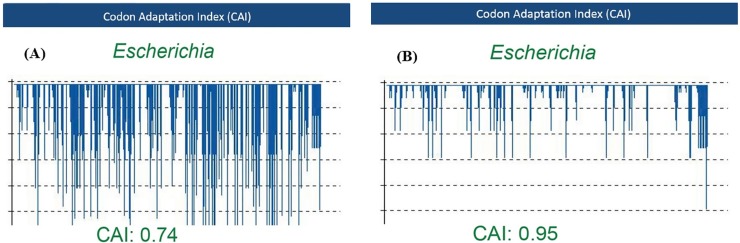
The value of Codon Adaptation Index (CAI) by GeneScript before and after optimization. (A) CAI index was calculated by GeneScript rare codon analysis tool to be 0.74 before codon optimization. (B) The CAI value was analyzed by GeneScript rare codon analysis tool to be 0.95 after codon optimization.

Codon optimization of the desired gene was conducted, using OPTIMIZER according to *E*. *coli* codon usage table provided by Kazusa codon usage database. The CAI of codon-optimized and non-optimized recombinant gene was analyzed by both OPTIMIZER and GeneScript rare codon analysis tool. The results showed a significant increase in CAI following codon optimization. CAI varies between 0–1. The genes with CAI values close to 1.0 and also G/C contents between 30% and 70% are predicted to be ideally expressed. The value of CAI analyzed by OPTIMIZER was 0.781 and 0.987 prior and subsequent to codon optimization, respectively. The value of this index was calculated by GeneScript rare codon analysis tool to be 0.74 and 0.95 prior and subsequent to codon optimization, respectively (Fig 2). The G/C content was also optimized to 42.94% by using OPTIMIZER. The CAI and G/C content values of the optimized *ompA-eltB* showed an enhanced probability of *ompA-eltB* high-yield expression in *E*. *coli*.

The sequence of recombinant *ompA-eltB* was designed by linking the 3’ end of a 396 bp region of *ompA*, which encodes loop 2–4 of *E*. *coli* O157:H7 OmpA, to 5`end of 311bp *eltB* gene (without signal sequence) via a sequence encoding of 6 amino acids as the linker sequence. The recombinant *ompA-eltB* was successfully synthesized and sub-cloned into a pET-24a (+) by Biomatik. The size and position of the synthesized construct was confirmed by double digestion, using XbaI and XhoI; in addition, the DNA sequencing was performed by Biomatik. A fragment of approximately 760bp was observed on the agarose gel, which was in agreement with the actual size of this fragment, i.e. 762bp. The DNA sequencing also validated 100% sequence homology between whole *ompA-eltB*-pET24a (+) and its designed nucleotide composition.

### Transformation and expression

To preserve the sufficient amount of recombinant gene, the *ompA-eltB*-pET24a (+) was transformed into *E*. *coli* DH5α. The size of the recombinant gene in the pET24a (+) was validated by performing PCR on the selected colonies of *E*. *coli* DH5α, using T7 promoter and terminator primers. The PCR products of approximately 900bp were observed after the 1% agarose gel electrophoresis, which were consistent with the expected size of 906bp of these products. The orientation of the recombinant gene was also validated through conducting the enzymatic digestion of *ompA-eltB*-pET24a (+) by using NdeI and XhoI. Following the digestion reactions, the linearized pET24a (+) and the excised fragments were observed on the 1% agarose gels.

**Fig 3:**
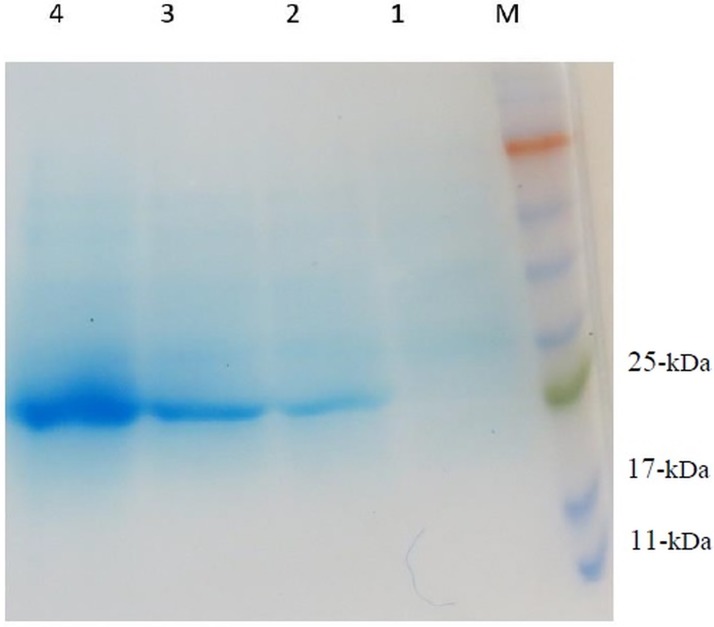
Expression pattern of recombinant OmpA-LTB. Coomassie-blue stained SDS-PAGE. Lane M: protein ladder. Lane 1: no induction of IPTG. Lanes 2, 3, and 4: recombinant protein induced with IPTG after 2, 4, and 24 hours with 27 KDa molecular weight.

**Fig 4:**
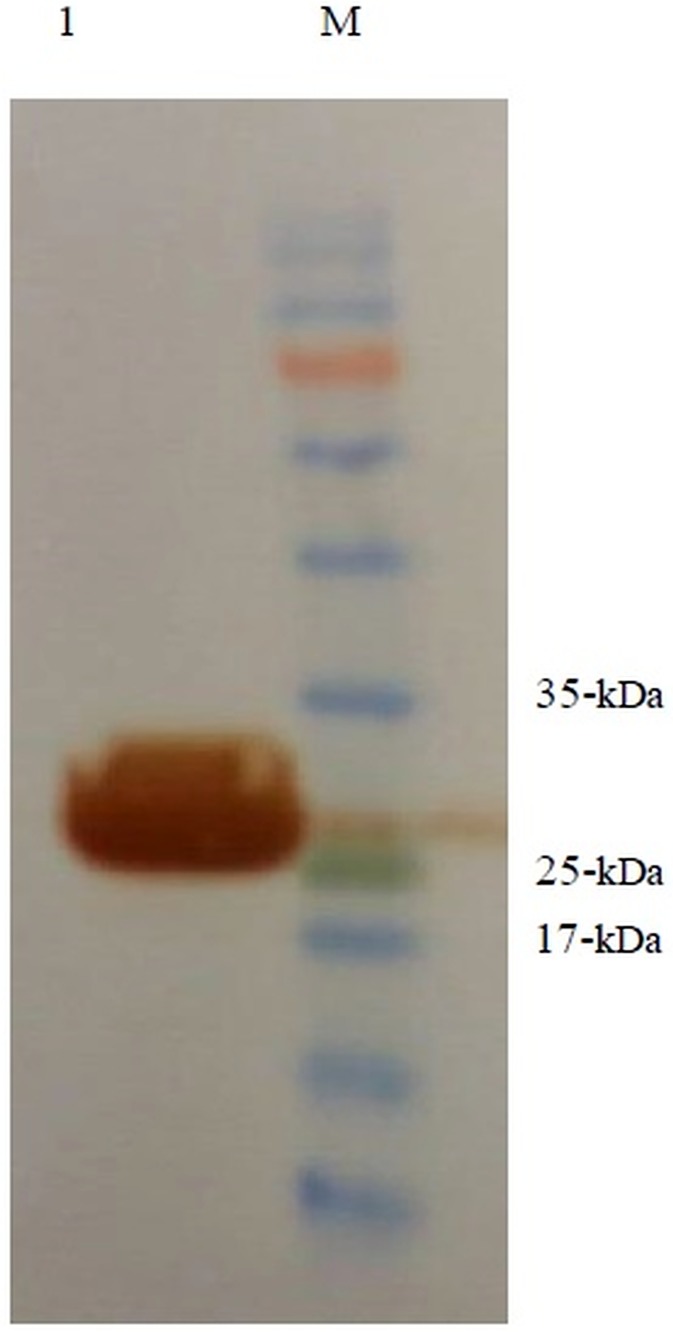
Western blot analysis of recombinant protein. Lane M: protein ladder. Lanes 1: recombinant OmpA-LTB protein with the same predicted size.

**Fig 5:**
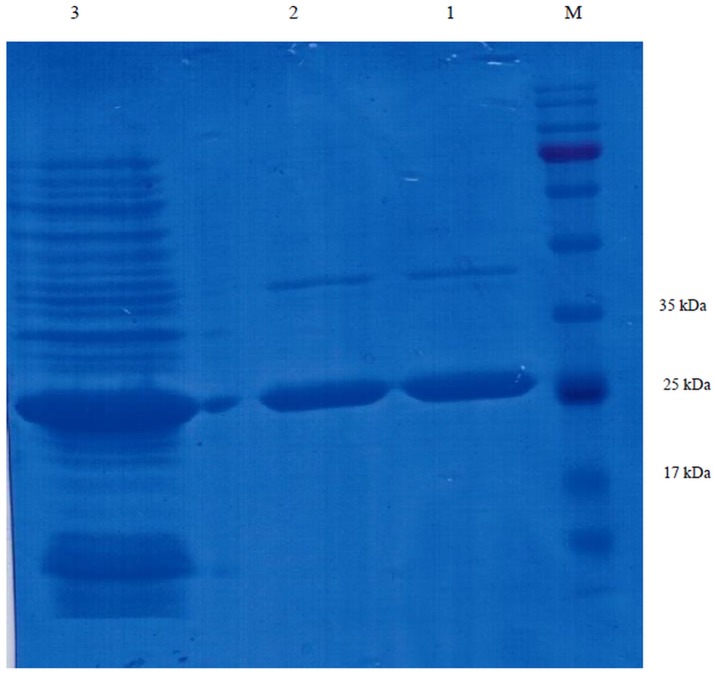
Purified recombinant OmpA-LTB protein with Ni-NTA column. Lane M: protein ladder. Lane 1 and 2: purified recombinant in elution buffer. Lane 3: recombinant protein before purification.

Subsequent to transforming the *ompA-eltB*-pET24a (+) into the *E*. *coli* BL21(DE3), single colonies were evaluated for potency of expressing OmpA-LTB after the induction of expression by IPTG. The result indicated that the amount of OmpA-LTB expression increased by enhancing the time of incubation post-induction so that the highest amount of the expressed protein was observed 24 hours after the induction by 1mM IPTG. The protein expression in whole cell lysates was confirmed by 12.5% SDS-PAGE; moreover, the highly expressed protein bands of 27kDa were observed in the samples which underwent induction by IPTG (Fig 3). Western blotting analysis was performed, using HRP conjugated mouse anti-polyhistdine monoclonal antibody to validate the identity of the expressed OmpA-LTB. The western blotting assay confirmed the expression of a protein about 27kDa, which is in concordance to the molecular weight of OmpA-LTB, 27619 Da (Fig 4). Owing to the 6X his-tag at the 3’ end of this chimeric protein, purification and isolation were carried out by Nickel affinity columns which caused no significant alteration in the conformation of protein domains [41–42]. The purification of highly expressed OmpA-LTB from cell lysate without major protein contamination was confirmed by 12.5% SDS-PAGE (Fig 5). Upon post-purification of SDS-PAGE, OmpA-LTB protein band was observed as an approximately 26–28 kDa protein, which is in concordance to the theoretically calculated molecular weight of this protein which is 27 kDa. The purification outcome led to the yield of 12 mg of purified protein from 1 L of *E*. *coli*.

### Dot-blot immunoassay

To ascertain the antigenicity of the recombinant OmpA-LTB protein, five BALB/c mice were injected subcutaneously by mentioned recombinant protein. The first and second dose of antigen were emulsified in equal amounts of Freund’s complete adjuvant and Freund’s incomplete adjuvant respectively with 10 days interval. 11 days after the last injection, sera were isolated and used as primary antibody to attach on OmpA-LTB dotted nitrocellulose membrane using dot-blot immunoassay. Dot-blot was used as a rapid and sensitive method to detect florescent signal which means antigen, OmpA-LTB, and antibody, Mouse IgG, interaction. The fluorescent signals under U.V light indicated presence of specific mouse IgG antibody against OmpA-LTB chimeric protein and confirmed antigenicity characteristic of the recombinant protein.

Participation of *E*. *coli* heat labile enterotoxin B subunit, as a potent adjuvant and suitable gastrointestinal protein carrier, in the OmpA-LTB structure will enhance the immunogenicity of this chimeric protein. LTB is a specific ligand of GM1 surface receptor on mammalian intestinal epithelial cells and Peyer’s Patches. This protein is capable of delivering antigens attached to it to the mentioned targets, making the attached antigens accessible to the immune cells at the mesenteric lymphoid tissues. Several reports indicate the capability of LTB to augment the immune response, especially mucosal antibody response. Therefore, LTB is considered as an appropriate biological adjuvant which has been frequently employed in vaccine development [13, 27, 43–45]. Then, upon oral uptake of OmpA-LTB, this protein is expected to accumulate on the surfaces of the intestinal epithelial cells and to be translocated into the mesenteric lymphoid tissue. Exposing this protein to innate and adoptive immune cells will trigger broad spectrum immune response to OmpA and probably LTB. As yet, no report of chimeric OmpA protein has been recorded; moreover, to the best of our knowledge, OmpA has not been utilized along with LTB or its components for any purposes.

This study described the successful expression and high-yield purification of a newly designed OmpA-LTB chimeric protein which can be considered as a potential human vaccine against *E*. *coli* O157:H7 infections. In-vitro and in-vivo evaluation of immune response to this protein is proposed to determine the capability of this protein as a vaccine against the above-mentioned infections.
